# How to Continue? New Approaches to Investigating the Effects of Adaptive Math Learning Programs on Students’ Performance, Self-Concept, and Anxiety

**DOI:** 10.3390/jintelligence11060108

**Published:** 2023-06-01

**Authors:** Anna Hilz, Karin Guill, Janina Roloff, Daniel Sommerhoff, Karen Aldrup

**Affiliations:** 1 Department of Educational Research and Educational Psychology, IPN—Leibniz Institute for Science and Mathematics Education, Olshausenstr. 62, 24118 Kiel, Germany; 2 Department of Mathematics Education, IPN—Leibniz Institute for Science and Mathematics Education, 24118 Kiel, Germany

**Keywords:** digital game-based learning, math learning program, intervention study, practice behavior, math performance

## Abstract

Math learning programs were expected to revolutionize students’ learning, but their effects so far have mostly been disappointing. Following the debate about *why* to continue research on math learning programs, we aimed to reformulate this question into *how* to continue this research. Investigations to date have neither considered a sufficiently wide set of outcome variables nor differentiated between performance measures (e.g., measuring addition and subtraction performance separately) and affective-motivational variables. Moreover, as students can only benefit from a program if they use it, researchers need to take practice behavior into account. Thus, we investigated whether the adaptive arithmetic learning program Math Garden fostered students’ addition and subtraction performance, their math self-concept, and a reduction of their math anxiety. We also investigated how practice behavior (practiced tasks/weeks) affected these outcomes. We used a randomized pretest-posttest control group design with 376 fifth-grade students in Germany. Students in the experimental condition practiced with Math Garden for 20.7 weeks and had an increase in math self-concept. The more subtraction tasks the students practiced, the more they improved their subtraction performance. We found no effects on math anxiety. The results are discussed in terms of providing a starting point for new directions in future research.

## 1. Introduction

It has always been a challenge for math teachers to provide instruction that meets the needs of all of their students, as students substantially differ in their prior math knowledge (Reinhold et al. 2019). This applies particularly to the transition from elementary school to secondary school (i.e., Grade 5 in most parts of Germany) when students from different elementary schools are regrouped into new secondary school classes. As the performance pressure in secondary schools increases, math teachers often do not have the time to address prior deficits and unlock students’ full potential regarding basic arithmetic operations. Hence, especially students who have problems understanding basic arithmetic operations are at risk of falling behind because basic arithmetic skills form the basis of understanding more complex mathematics (Andersson 2010; Bailey et al. 2014; Hansen et al. 2017; Jordan et al. 2013) and are a strong predictor of later achievement (Bailey et al. 2012; Barbieri et al. 2021; Duncan et al. 2007; Siegler et al. 2012).

Math learning programs may help to meet this challenge due to their beneficial features, such as adaptivity and corrective feedback. They have, therefore, moved into the focus of research in recent decades (Higgins et al. 2019; Hillmayr et al. 2020; Pellegrini et al. 2021; Sailer and Homner 2020). Unfortunately, the results obtained up until now regarding achievement outcomes are disappointing. In their meta-analysis examining the overall effect size of digital game-based learning, Byun and Joung (2018) found a statistically significant but only small effect size (*d* = 0.37). Hence, they concluded that there may be other ways for students to learn math more effectively (Byun and Joung 2018). Similarly, in their meta-analysis, Tokac et al. (2019) found only a small, marginally significant overall effect (*d* = 0.13) for game-based interventions in math compared to traditional nonvideo game-based classroom instruction, and the authors also pointed out the high heterogeneity regarding the effectiveness of such game-based interventions. Pellegrini et al. (2021), who also found only a very small nonsignificant effect size (*d* = 0.05) concerning the implementation of digital technology in math instruction, sharpened the topic by pointing out that the use of technology in education has been expected to have revolutionary impacts on learning outcomes but that, no matter how beneficial and promising program mechanisms sound, they have not yet improved students’ performance substantially. But what is the educational implication of these disappointing results? Should learning programs no longer be recommended for use in the classroom because research is not able to show that their implementation is effective? Rather than assuming that math learning programs are ineffective and potentially rejecting their use, one could take a step back and ask whether the methodological approaches of previous studies were fully capable of detecting potential effects. Thus, regarding the question of *why* to continue research on math learning programs, we suggest this question is reformulated into *how* to continue research on math learning programs. Posing this question is highly relevant because prematurely rejecting the use of math learning programs might lead to these interventions being abandoned, even though they are easily applicable and cost-effective and can thus reach large numbers of students.

Thus, the present study identified current desiderata in research on math learning programs, addressed them conceptually, and then analyzed the effects of an adaptive computer-based arithmetic learning program with a randomized pretest-posttest control group design in a sample of fifth-grade students in Germany (*N* = 376). The approaches postulated in this study might be a starting point for future research on math learning programs.

### 1.1. Promising Mechanisms of Math Learning Programs

The central promising characteristics of math learning programs are their adaptivity and corrective feedback (Hillmayr et al. 2020). Adaptivity in this context means that the task difficulty adapts to the individual ability level of each learner (Hillmayr et al. 2020), while corrective feedback highlights correct solutions and corrects incorrect solutions (Shute 2008). There is a wide variety of interactive math learning programs that include these desirable mechanisms and that have also already been used for scientific purposes, for example, Bettermarks from Germany, Mindsteps from Switzerland, or Carnegie Learning from the U.S., but they all differ in their conceptual functioning (Hillmayr et al. 2020).

In this study, we used the adaptively working arithmetic learning program Math Garden (Klinkenberg et al. 2011; Straatemeier 2014). Following the characterization by Nattland and Kerres (2009), Math Garden can be classified as a drill-and-practice program. Drill-and-practice programs serve to strengthen previously acquired content knowledge by allowing the learner to practice at their own pace and to repeat specific types of exercises as often as necessary (Nattland and Kerres 2009). In line with this definition, Math Garden allows students to strengthen basic arithmetic operations that are central to elementary school curricula on individual performance levels because an algorithm regulates the assignment of the tasks so that students have an average percentage of correct tasks of 75% (Klinkenberg et al. 2011). Moreover, after each task, direct corrective feedback is given. The interplay of these promising mechanisms might affect not only students’ performance but also their affective-motivational outcomes, such as math self-concept and math anxiety, as further elaborated below.

#### 1.1.1. Effect Mechanisms of Math Learning Programs on Math Performance

The cognitive theory of multimedia learning (Mayer 2014) describes why using digital learning programs such as Math Garden can be beneficial for students’ performance: Students need to actively engage with the learning content in order to understand new information (Mayer 2014). Hence, interactive digital learning programs help them to directly influence their own learning processes, as the defining feature of interactivity is responsiveness to the learner’s actions during learning (Moreno and Mayer 2007). Thus, programs such as Math Garden respond to students’ ability level by providing appropriate tasks, and immediate program feedback helps students to instantly reflect on their performance and, if necessary, to rethink inefficient calculation strategies (Shute 2008). Besides performance, similar effect mechanisms might also affect math self-concept.

#### 1.1.2. Effect Mechanisms of Math Learning Programs on Math Self-Concept

Math self-concept refers to the self-evaluation of one’s ability in math and is considered to be an important factor concerning performance and achievement-related choices (Eccles and Wigfield 2020). According to the reciprocal effects model (Marsh 1990; Marsh and Martin 2011), performance and math self-concept enhance each other. This has been confirmed by several studies (Arens et al. 2022; Jiang et al. 2020). As Math Garden offers high success rates independent of students’ actual skill level because problems are adapted to the individual skill level during practice (Klinkenberg et al. 2011), all students can be expected to experience positive feedback and rewards, which then should imply good performance. Thus, they might perceive their own abilities as being high, generalize this positive feedback to an entire subject area (e.g., math), and internalize it into a positive self-concept for that subject area (Craven et al. 1991). Hence, receiving feedback that they have solved math tasks successfully should lead to an increase in students’ math self-concept. In addition to math self-concept, math anxiety is another prominent affective-motivational construct that has been shown to have a direct relation to students’ performance (Ashcraft and Kirk 2001; Caviola et al. 2022; Devine et al. 2018; Ma 1999; Pekrun 2006).

#### 1.1.3. Effect Mechanisms of Math Learning Programs on Math Anxiety

Richardson and Suinn (1972) defined math anxiety as a “feeling of tension and apprehension that interferes with the manipulation of numbers and the solving of math problems in a wide variety of ordinary life and academic situations” (p. 551). The control-value theory (Pekrun 2006) postulates that (math) anxiety occurs when the value of an achievement-related task is perceived as high but solving the task is considered to be uncontrollable due to missing resources, such as a lack of knowledge and skills. However, as adaptive programs such as Math Garden adjust the task difficulty to the learner’s skill level, students with math anxiety should perceive the tasks as being (more) controllable in terms of solving the tasks correctly, which should reduce their anxiety. Positive program feedback should help students to recognize the controllability of tasks even further. Moreover, programs such as Math Garden offer students the chance to make mistakes in private. As negative experiences with math in public appear to contribute to math anxiety (Bekdemir 2010), students should feel safer making mistakes during practice with a math learning program than during oral practice sessions with the whole class. Hence, if the number of public, potentially negative, or embarrassing math-related experiences is reduced, students might experience even further control.

Nonetheless, even though the use of arithmetic learning programs like Math Garden in school as an additional instructional feature to strengthen arithmetic skills and to foster affective-motivational outcomes is promising in theory, researchers often detected no effects or only small effects on math performance (Bai et al. 2012; Byun and Joung 2018; Hung et al. 2014; Jansen et al. 2013; Pareto et al. 2012; Pellegrini et al. 2021; Tokac et al. 2019). In contrast to performance effects, affective-motivational effects have not been investigated much so far. Hence, we considered whether a change in research on math learning programs might be necessary to improve the quality of studies in this field. First studies have already started to investigate math learning programs in a more differentiated way. For instance, Hassler Hallstedt et al. (2018) investigated addition and subtraction performance separately, Jansen et al. (2013) considered not only performance as an outcome but also affective-motivational variables to examine the effectiveness of math learning programs while also taking practice behavior into account, and Vanbecelaere et al. (2022) also asked for a systematic and more standardized assessment framework of digital games for learning. Our aim was to build upon these studies and combine their methodologies in one approach.

### 1.2. New Approaches to Investigating the Effectiveness of Math Learning Programs

When considering how future studies on math learning programs can be improved, and after reviewing the literature on math learning program evaluations, in our view, three different approaches can be taken to investigate the effectiveness of math learning programs in new ways.

#### 1.2.1. Measuring Distinct Subdomain Performance

As a key learning outcome, researchers mostly aim to measure students’ math performance improvement after implementing a math learning program. To do so, they usually assess performance with a broad status diagnostic score including domains such as measurement, geometry, or arithmetic (Bai et al. 2012; Beserra et al. 2014; Ke 2008; Shin et al. 2012). However, such an overall performance score may not be able to reveal improvements in more specific math subdomains, such as different arithmetic operations (e.g., addition or subtraction). Hence, Ran et al. (2021) called for more specific outcomes when investigating math learning programs, arguing that an overall score might lead to flawed conclusions about the effectiveness of the intervention. For instance, a significant performance improvement as shown in an overall arithmetic score does not necessarily imply an equally large performance improvement in addition and subtraction, whereas, in turn, no performance in an overall arithmetic score does not necessarily mean that there is no improvement in addition and subtraction. To the best of our knowledge, so far, only Hassler Hallstedt et al. (2018) differentiated between single performance measures for addition and subtraction skills in their analyses. They were able to show that a math tablet intervention had effects on low-achieving second graders. They found a performance increase in both addition and subtraction (Hassler Hallstedt et al. 2018). This underlines the importance of using distinct performance measures, as the study clearly showed that the intervention was effective for both addition and subtraction. Hence, more research that differentiates between performance measures is needed to gain a more detailed insight into which exact math abilities are fostered with the learning program being implemented. Such research would probably reveal hidden effects.

#### 1.2.2. Affective-Motivational Outcomes

In addition to the differentiated effects on performance that have not been sufficiently researched thus far, the effects on affective-motivational outcomes have also been mostly neglected. Given that math self-concept and math anxiety are essentially related to students’ math performance and their engagement in math (Ashcraft and Krause 2007; Chinn 2009; Guay et al. 2003; Marsh and Martin 2011), a learning program, even if it does not increase performance in the first step, can be effective in the long term by improving math self-concept or reducing math anxiety. Indeed, the results of a meta-analysis by Fadda et al. (2022), which is, to the best of our knowledge, the only existing meta-analysis regarding the motivational effects of math learning programs, showed that providing math learning programs can foster students’ motivation. The authors pointed out that the highest effects were found in studies that operationalized motivation as expectancy of success and related beliefs about competence (i.e., self-concept) in contrast to value components (Fadda et al. 2022). However, as only three studies were of high methodological quality and met the criteria to be included in the meta-analysis regarding the effects of math self-concept, more research is needed here. Further, only very few studies so far have focused on the effects of math learning programs on the reduction of math anxiety. In their intervention study, Huang et al. (2014) showed a decrease in math anxiety from pretest to posttest, whereas Hung et al. (2014) did not find any effects of their intervention on math anxiety. However, both studies only had a small experimental sample, with 25 students each per condition. Moreover, the studies differed substantially in their number of treatments during the intervention: Huang et al. (2014) had 40-min sessions twice a week over six weeks, therefore, this was a more distributed intervention, whereas, in contrast, Hung et al. (2014) only had one 240-min session. Hence, this was a more massed intervention. These results might indicate that a more distributed intervention over a longer period of time is needed to reduce math anxiety, as students probably need some time to perceive tasks as being controllable. However, the authors only supervised the practice sessions and did not control for the number of tasks that were actually practiced during their intervention. This lack of data brings us to the last desideratum.

#### 1.2.3. Considering Practice Behavior

Many studies only provided students with a learning program and then evaluated whether such an intervention made a difference in the performance of this group compared to a wait-list control group (Bai et al. 2012; Chang et al. 2015; Huang et al. 2014; Hung et al. 2014; Shin et al. 2012). This approach neglects the actual practice behavior of the students. However, as researchers have already shown in meta-analyses that short-term math learning program interventions lead to higher effectiveness, due to higher student engagement at the beginning of the intervention (novelty effect) than long-term interventions (Hillmayr et al. 2020; Sung et al. 2017), practice behavior needs to be considered.

The body of literature on practice behavior shows that students need to practice regularly over a longer period of time to achieve the best performance outcomes (Barzagar Nazari and Ebersbach 2019; Carpenter et al. 2012; Vlach and Sandhofer 2012). Thus, it is reasonable to assume that a math learning program may fail to have effects because students simply practiced too little with the program. This assumption cannot be tested simply by investigating the effects of the provision of a learning program. Indeed, some experimental studies supervised students’ practice sessions with a math learning program in classrooms to ensure that they practiced a given number of tasks (Huang et al. 2014; Rodrigo 2011; Shin et al. 2012; van den Heuvel-Panhuizen et al. 2013), and all were able to show student performance gains. However, this approach can only provide limited information about how students interact with the program when they are not supervised, not only in school but, for instance, also at home. With regard to increasing digitalization and the possibility of using log and trace data, researchers should take advantage of this potential to uncover hidden behavioral patterns in a more naturalistic setting (Baker and Inventado 2014; Haleva et al. 2021). Unfortunately, the number of studies that took log and trace data into account is still rather modest. The few studies referring to such data mostly considered the time spent practicing with math learning programs (Haleva et al. 2021; Hassler Hallstedt et al. 2018; Louw et al. 2008; Spitzer 2022), and they all were able to show that time spent practicing improved student performance. Another approach that can be employed to take practice behavior into account is to consider the number of tasks practiced by the students in the program. This is more of an in-depth approach in contrast to the time spent practicing as, for example, students can solve 10 tasks in 10 min if they concentrate but can solve only two tasks in the same 10 min if they are distracted. Jansen et al. (2013) showed that performance improvement in the program Math Garden was mediated by the number of tasks attempted. The authors also considered practice behavior in terms of practiced tasks when investigating effects on motivation-related variables (i.e., perceived math competence) and math anxiety, but they did not find any effects (Jansen et al. 2013). This might be due to practiced tasks not being the optimal indicator to show the effects of practice behavior on affective-motivational variables. Another alternative, which has not yet been considered in research, would be to include the number of weeks over which students practiced with the program as practice behavior (referred to from here on as “practiced weeks”). As the practiced tasks are more a measure of quantity, the practiced weeks better reflect how regularly students practiced with a program (i.e., distributed practice behavior). As previous studies have already shown that students benefit most from math learning opportunities if their practice behavior is distributed (Barzagar Nazari and Ebersbach 2019; Schutte et al. 2015), including the number of practiced weeks could represent a new and valuable facet of practice behavior.

### 1.3. The Present Study

This study addresses three research desiderata on math learning programs and provides empirical data that can be used for future investigations. First, we used distinct arithmetic performance measures (i.e., addition and subtraction) to investigate potential performance increases on a detailed level. Second, we considered affective-motivational outcomes (i.e., math self-concept and math anxiety) to investigate in which different ways students may benefit from the implementation of Math Garden. Third, we took practice behavior in terms of practiced tasks and practiced weeks in Math Garden for all outcomes into account (i.e., performance and affective-motivational variables), as missing effects might occur because students practiced too little. This resulted in three research questions regarding the provision of Math Garden and students’ actual practice behavior in terms of practiced tasks and practiced weeks:

How does the provision of Math Garden affect students’ addition and subtraction performance, their math self-concept, and their math anxiety? On the basis of the theoretically assumed promising program features, we expected that the provision of Math Garden would improve (H1.1) students’ addition and (H1.2) subtraction performance. We assumed that the program would additionally (H1.3) increase students’ math self-concept and (H1.4) decrease students’ math anxiety.

How does the number of addition and subtraction tasks practiced affect students’ addition and subtraction performance, respectively? How does the number of all math-related tasks (including addition, subtraction, and other types of math tasks, see Section 2.3.4) practiced in Math Garden affect students’ math self-concept and their math anxiety? We expected that (H2.1) the more students practiced addition tasks during the intervention, the higher their addition performance after the intervention would be. We expected that (H2.2) the more students practiced subtraction tasks, the higher their subtraction performance would be. Moreover, we expected that students who practiced more math-related tasks overall would have (H2.3) a higher math self-concept and (H2.4) lower math anxiety after the intervention because they received more positive feedback due to the adaptive algorithm.

How does the number of practiced weeks spent on addition and subtraction tasks (referred to from here on as “practiced weeks of addition or of subtraction”) affect students’ addition and subtraction performance, respectively? How does the total number of practiced weeks (including weeks spent practicing addition, subtraction, and other types of math tasks, see Section 2.3.4) in Math Garden affect students’ math self-concept and their math anxiety? We expected that (H3.1) students who spent more weeks practicing addition would have higher addition performance. We expected that (H3.2) students who spent more weeks practicing subtraction would have higher subtraction performance. Moreover, we expected that students who spent more weeks practicing with Math Garden overall would have (H3.3) a higher math self-concept and (H3.4) lower math anxiety after the intervention.

We addressed our research questions and hypotheses with a pretest-posttest control group design with a random assignment of students on the class level, assessing the suggested student outcomes with objective measures outside of Math Garden, which is a major strength of this study. The time period spent practicing with the program in our study was substantial (*M* = 20.7 weeks), and we had a large sample (*N* = 376) with similar numbers of students in both groups. We chose to use Math Garden because it is a low-cost program that was designed to be easily implemented in schools and to track students’ practice behavior. To ensure the robustness of our findings, we considered several important control variables in our analyses. We controlled for students’ pretest scores to account for differences in their baseline levels. Moreover, we controlled for students’ gender and a possible migration background because previous studies showed that these variables had effects on our outcome variables (Liu and Wilson 2009; Marsh and Yeung 1998; van Mier et al. 2018). As addition and subtraction performance was measured on tablets in a speed test, we also checked for typing speed (Hassler Hallstedt et al. 2018).

## 2. Materials and Methods

### 2.1. Design and Procedure

Student data were collected in schools from October 2019 (T_1_) to March 2020 (T_2_) in compliance with data protection requirements, and the study was approved by the institutional ethics committee. Participation in the study was voluntary and parental permission was obtained before students participated in the study.

The participating classes completed math performance tests and answered questionnaires assessing the relevant variables of interest for our study. A testing session lasted approximately 1.5 h and was done with tablets. All students were tested within a three-week period. At the end of the initial testing session, the classes were randomly assigned either to an experimental group or to a wait-list control group. Hence, variance differences between schools were controlled for. The students in the experimental group received a 10-min introduction to Math Garden and accessed the program on tablets using personalized login data provided by their teachers. While practicing, they got a brief oral introduction to the main program principles. At the end of the introductory practice session, students were told that they were allowed to, and should, practice with the program at home and in class on available digital devices (e.g., desktop computer, tablet, or smartphone). Their teacher also received a short written introduction to Math Garden and was asked to integrate the program into regular math lessons, to assign it for homework, or to use it during supervision classes, which provided him or her with many opportunities to individually adapt the use of Math Garden to meet all students’ needs. Students could access the program for, on average, 20.7 weeks. Their weekly practice behavior was tracked. Three classes did not have personal login data during the initial testing session due to technical problems. These students were introduced to the program in a 5-min presentation. The teachers were asked to introduce the students to the program in more detail later. In March 2020, after 22 weeks, the classes were tested again, and the students answered questionnaires. After this testing session, the wait-list control group received access to Math Garden.

### 2.2. Sample

The data for this study were obtained from a sample of 376 fifth graders from 21 classes at seven urban comprehensive (non-academic-track) schools in northern Germany. While academic-track schools in Germany prepare students primarily for higher education, non-academic-track schools offer different levels of school degrees (Maaz et al. 2008).

We excluded one student due to an implausibly high level of practice (more than 2000 tasks within one week), which might have been caused by siblings also practicing with the program, and we excluded another five students because they had missing data on all relevant variables, such as questionnaire data and practice data. The final sample then consisted of 200 students in the experimental condition (50.0% male, 53.4% with a migration background) and 170 students in the wait-list control condition (52.7% male, 43.2% with a migration background). The two conditions did not differ significantly in their pretest mean values (see Table 1). A priori power analyses (Faul et al. 2007, 2009) showed that for between-group analyses (Research Question 1), setting the estimated effect size *d* to 0.30 and assuming a power of at least .80 with an α-level of .05, a sample size of 176 students per group would be needed. Further, for within-group analyses (Research Questions 2 and 3), setting the estimated effect of *R*^2^ to .08, assuming a power of at least .80 with an α-level of .05, a sample size of 154 students would be needed. Unfortunately, the posttest coincided with the COVID-19 lockdown in March 2020 in eight classes, which led to missing data on all posttest scores for 66 (33%) students in the experimental condition and for 50 (29%) students in the wait-list control condition. We did not exclude these students from our analytic sample (see Section 2.4 for missing data handling).

### 2.3. Measures

#### 2.3.1. Addition and Subtraction Performance

We used the addition and subtraction scales of the Heidelberg calculation test by Haffner et al. (2005) to measure math performance. Each scale consists of 40 items that become increasingly difficult. Students are instructed to solve as many items as possible in 120 s. Thus, this test also assesses quick mental arithmetic, which is exactly what students were asked to practice in Math Garden. As our goal was to measure changes in student performance, we expected most performance improvement to occur on items with high item-scale correlations. However, due to the conceptual design of the Heidelberg calculation test, including all items might systematically underestimate the predictive power of the change diagnostics because the first items may cause ceiling effects, and a speed component may affect the last items, which could result in these items then having low item-scale correlations. Therefore, we decided to make an item selection, and we only considered items that had an item-scale correlation higher than .50 (Item 14 to 24 for the addition scale and Item 15 to 25 for the subtraction scale) for performance scores. We found good retest reliability values for the addition (*r_tt_* = .52) and subtraction scales (*r_tt_* = .62). To ensure transparency, we additionally ran our analyses with items that had an item-scale correlation higher than .30 and with all items that the students completed.

#### 2.3.2. Math Self-Concept

Math self-concept was measured with a scale adapted by Köller et al. (2000). The scale consists of five items (e.g., “I would prefer to do math if the subject was not that difficult.”), which have to be answered using a four-point Likert scale (1 = does not apply at all to 4 = fully applies). All items were reverse coded so that high scores represent a high math self-concept. Cronbach’s alpha was .83 in the pretest and .84 in the posttest. Further, the pre- and posttest scores correlated significantly (*r_tt_* = .64).

#### 2.3.3. Math Anxiety

Math anxiety was measured with a subscale from the Math Anxiety Rating Scale for Fourth to Sixth Grades by Roick et al. (2013). The scale consists of five items (e.g., “How nervous do you feel when a math test is written?”), which have to be answered using a five-point Likert scale (1 = not nervous at all to 5 = very nervous). Cronbach’s alpha was .90 in the pretest and .92 in the posttest. The pre- and posttest scores here were also significantly correlated (*r_tt_* = .37).

#### 2.3.4. Practice Behavior with Math Garden

Math Garden is an adaptively working web-based learning program. The adaptive algorithm is based on the Elo (1978) rating system, which allows for on-the-fly estimation of item difficulty and person ability parameters (for further information, see Klinkenberg et al. 2011). After solving a task correctly, students receive positive feedback and are presented with a new task with a similar or slightly higher difficulty level. After solving a task incorrectly, students receive corrective feedback and are presented with a new task with a similar or slightly lower difficulty level. Students cannot repeat previous tasks.

Math Garden automatically tracks the number of tasks completed per domain per week (log files). Thus, for each student, we estimated six practice frequency scores: We calculated the number of tasks completed in the domains of addition and subtraction, respectively. We calculated the number of weeks for which students practiced addition tasks and subtraction tasks, respectively. We calculated an overall practice frequency score by calculating the total number of math-related tasks completed in Math Garden, which included tasks on addition, subtraction, multiplication, division, mix (mixture of basic arithmetic operations), counting (of objects), series (logical completion of a given number series), numerals (tasks in which numbers must be combined to obtain a desired target number), and tables (multiplication tables tasks from 1 to 10), and we calculated the total number of practiced weeks for all math-related tasks for each student. The descriptives for these six different scores are reported in Table 2.

We had a maximum observation period of 22 calendar weeks (Calendar Week 43 in 2019 to Calendar Week 12 in 2020). The observation period varied for two reasons: First, single classes were tested (i.e., pretest and questionnaire) over a period of three weeks at the beginning of the study (for logistical reasons), and second, three teachers did not provide the login data to their classes at the start of our study. On average, the program was available to the students for 20.7 weeks (*SD* = 1.61, *min* = 17, *max* = 22).

#### 2.3.5. Covariates

Students’ gender was coded as 0 = male and 1 = female. A migration background was assessed with the following item: “Are other languages spoken at home besides German?” with 0 = no and 1 = yes as the answer options (Happ et al. 2021). The speed scale of the Heidelberg calculation test (Haffner et al. 2005) was used to control for how fast students were at tapping on tablets.

### 2.4. Data Analysis

To answer our research questions, we ran a total of 12 multiple regression analyses in M*plus* (Version 8.5; Muthén and Muthén 2008–2020). Addressing the question of how the provision of Math Garden affects addition and subtraction performance, math self-concept, and math anxiety, the predictor was the group assignment (0 = wait-list control group, 1 = experimental group, four multiple regression models). This dummy-coded variable makes it possible to statistically test mean differences between the different groups (Cohen et al. 2003). The dependent variables were the posttest scores when controlling for pretest scores and the other covariates. We additionally calculated effect sizes (Cohen’s *d*_z_) to estimate the size of the mean differences.

To investigate the degree to which practice behavior with Math Garden affected our outcomes, we only analyzed the experimental group, as the wait-list control group did not generate any practice data in Math Garden. The dependent variables were the posttest scores when controlling for pretest scores and other covariates, but the predictors were either the number of practiced tasks (Research Question 2, four multiple regression models) or the number of practiced weeks (Research Question 3, four multiple regression models). However, due to our two distinct subdomain performance measures (i.e., addition and subtraction), we made further differentiations: We used the number of addition tasks practiced/practiced weeks of addition to predict addition performance and the number of subtraction tasks practiced/practiced weeks of subtraction to predict subtraction performance. As we assumed that the effects on math self-concept and math anxiety would be less related to the practicing of specific tasks (e.g., addition or subtraction) and more related to feedback mechanisms such as the adaptive algorithm, we used the number of all tasks practiced/all weeks practiced in Math Garden to predict math self-concept and math anxiety, respectively.

As we made an item selection (item-scale correlation > .50) for the performance measures, we additionally ran robustness analyses for all of the models that included performance measures. For this, we calculated the effects on performance again with items that had item-scale correlations higher than .30 (Item 9 to 28 for the addition scale and Item 11 to 30 for the subtraction scale) and with all items answered by the students. These results are provided in the online material (see Tables S1–S4).

Because students were nested within classes, we took the two-level nature of the data into account by using the TYPE = COMPLEX command. The COMPLEX command adjusts standard errors for nonindependence within classes. Therefore, nonindependence within classes was accounted for but not explicitly modeled. Further, we used the robust maximum likelihood estimator to obtain standard errors that were robust to nonnormality (e.g., Bandalos 2014; Maydeu-Olivares 2017). To ease the interpretation, all continuous independent variables were *z*-standardized (*M* = 0, *SD* = 1).

In many empirical studies, missing data are a potential methodological problem. We found missing data on all variables. Regarding the self-reported and test measures, this was because some students in our final analytic sample were absent during at least one of both testing sessions (*N* = 156), for different reasons. On the one hand, dropouts resulted in the following missing data pattern: Four students participated in neither the pretest nor the posttest, but they provided Math Garden data, 10 students did not participate in the pretest but did participate in the posttest, and 26 students participated in the pretest but did not participate in the posttest. On the other hand, the posttest coincided with the COVID-19 lockdown in March 2020 in eight classes, which led to further missing data on all posttest scores for 66 (33%) students in the experimental condition and for 50 (29%) students in the wait-list control condition, although we tried to collect information on the self-reported measures with an additional online survey. Statistical comparisons between the group of students with missing data and the group of students without missing data are provided in the online material (see Table S5). The results show that the two groups did not differ regarding their self-reported measures and performance test, but the group of students with missing data had lower practice behavior scores, except for the addition domain. Moreover, three students participated in at least one testing session and received login data, but it was not possible to match their responses from the testing session to their practice data, which then resulted in missing practice data. Overall, the percentage of missing data varied from 0% on group assignment to 41.6% on math self-concept (T_2_), with an average missing rate of 11.6%. To handle the missing data, we used the full information maximum likelihood approach, which means that missing values were not imputed or filled in, but model parameters and standard errors were directly estimated using all available raw data while no data points were excluded (Enders 2001, 2022). Thus, we avoided listwise deletion of students with missing data on single measurement occasions.

## 3. Results

### 3.1. Descriptives and Preliminary Analyses

The means and standard deviations for the self-report and performance measures are reported in Table 1. *t*-tests revealed that pretest scores and the measured covariates did not significantly differ between the two conditions, which suggests that the randomization on the class level worked well. Regarding our relevant study variables, we noticed that, on average, math self-concept scores descriptively dropped from pre- to posttest in the wait-list control condition, but we found an increase in the experimental condition. The mean values for the performance measures descriptively increased in both conditions, and anxiety scores slightly decreased on average in both conditions from pre- to posttest.

Table 2 displays the descriptives of practice behavior in Math Garden. The high variance of practiced tasks over the 22-week period is noticeable. Overall, students practiced, on average, for 4.62 weeks (*SD* = 2.96) with Math Garden, with a minimum of one week and a maximum of 13 weeks.

Table 3 and Table 4 show the correlations between the independent variables and the dependent variables for all students and for the experimental group only. Regarding the possible effects of Math Garden on our outcome variables, we found significant correlations for group assignment with math self-concept at T_2_ (*r* = .12, *p* = .031; see Table 3).

### 3.2. Effects of Providing Math Garden

Our first research question concerned differences between pre- and posttest scores concerning addition and subtraction performance, math self-concept, and math anxiety between the wait-list control group and the experimental group. After controlling for gender, a possible migration background, and tablet typing speed, we found a statistically significant effect of providing Math Garden on students’ math self-concept (see Table 5). As expected (H1.3), providing students with Math Garden fostered their math self-concept in contrast to the wait-list control condition (β = .12, *p* = .002; *d*_z_ = 0.26). According to Cohen (1988), this effect size can be described as small. Concerning our Hypotheses H1.1, H1.2, and H1.4, we did not find any significant effects.

### 3.3. Effects of Practiced Tasks

To answer our second research question, we examined how the number of tasks practiced in Math Garden affected addition and subtraction performance, math self-concept, and math anxiety in the experimental group (see Table 6). After controlling for gender, a possible migration background, and tablet typing speed, we found a statistically significant effect of the number of subtraction tasks practiced on students’ subtraction performance. As expected (H2.2), the more subtraction tasks students practiced, the higher their subtraction performance was (β = .10, *p* = .008). Regarding Hypotheses H2.1, H2.3, and H2.4, we did not find any statistically significant effects.

### 3.4. Effects of Practiced Weeks

Addressing our third research question, we examined how the number of practiced weeks in Math Garden predicted addition and subtraction performance, math self-concept, and math anxiety in the experimental group (see Table 6). We did not find any statistically significant effects regarding our hypotheses (H3.1–H3.4) in the original analyses. However, in further analyses (the results of which are provided in the online material, see Table S4), we found—as expected—that the more weeks students spent practicing addition in Math Garden, the higher their addition performance in the posttest was (β = .13, *p* = .015). In these analyses, math addition performance was measured with items that only had item-scale correlations higher than .30. In the original analysis, this effect was marginally significant. We discuss the implications of this result in the following section.

## 4. Discussion

By addressing new approaches that can be taken to continue investigating math learning programs, the aim of this study was to explore the effects of the arithmetic learning program Math Garden on distinct subdomain performance measures and on affective-motivational outcomes (i.e., math self-concept and math anxiety), while also taking practice behavior in Math Garden into account. The results supported only some of our hypotheses. We were able to show that providing Math Garden fostered students’ math self-concept, in contrast to a wait-list control condition, and we also showed that the more subtraction tasks students practiced in Math Garden, the higher their subtraction performance in the posttest was. While the items selected for analysis did not show an effect of practiced weeks of addition on students’ addition performance in the posttest, an extension to include items with item-scale correlations higher than .30 indicated that the number of practiced weeks of addition was related to students’ addition performance. Our other hypotheses were not confirmed.

### 4.1. Effects of Math Garden on Math Performance

We did not find any effects of the mere provision of Math Garden on addition or subtraction performance. Given the ongoing debate about the effectiveness of math learning programs (Byun and Joung 2018; Pellegrini et al. 2021), this lack of performance effects is not entirely unexpected and again highlights the problem of heterogeneous research findings in this area (Tokac et al. 2019). However, by only investigating the effects of providing the program, many studies are limited at this point, and one can only speculate about why missing effects occur. One possible explanation for the missing results might be that Math Garden does not help students improve their addition and subtraction skills. However, as we would expect Math Garden to have only small effects on students’ learning because the program does not intend to teach students new content but rather to repeat what has already been learned, these expected small effects might be especially difficult to reveal if students have not practiced much with the program. Thus, an alternative possible explanation would be that students did not practice enough with the program to benefit from it, but to make any further suggestions about this, practice behavior needs to be taken into account. In our study, we considered two different types of practice behavior, namely, the number of practiced tasks and the number of practiced weeks. Whereas, for this study, the number of practiced tasks was primarily a measure of quantity, the number of practiced weeks might better reflect the quality of the practice, that is, how regularly students practiced with the program. We found an expected effect of the number of subtraction tasks practiced on subtraction performance, but we did not find an effect of practicing addition tasks on addition performance. In terms of the number of practiced weeks, we found an inverse pattern, that is, we did not find an effect of the number of practiced weeks of subtraction on subtraction performance, but we found an expected positive effect of the number of practiced weeks of addition on addition performance. However, this second effect only occurred when performance was measured with items that had item-scale correlations higher than .30. In contrast, the main analysis was conducted with items that had item-scale correlations higher than .50. Thus, this result needs to be interpreted with caution and can only be generalized to a very limited extent. The effects of subtraction tasks practiced on subtraction performance seem to be more robust because we found this effect in all of our analyses.

In general, according to our G*Power analyses (Faul et al. 2007, 2009), the missing effects of practice behavior might be explained by the reduction of sample size due to the COVID-19 school closures and, thus, a loss of power. Hence, for future research, we recommend that this study is replicated in a bigger sample. Further, inactivity and dropout rates are serious issues when investigating the effects of learning programs, and they might explain missing effects as well (Bacca-Acosta and Avila-Garzon 2021; Spitzer and Musslick 2021). Students might have shown high engagement with Math Garden right after receiving it, which, in turn, might have resulted in short-term performance increases, but they might then have become inactive in the long-term so that effects were no longer detectable, especially at the end of a longer intervention such as ours. Thus, on the one hand, we would like to emphasize the importance of investigating how to put students in a mindset where they continuously and systematically engage with such learning programs (Spitzer and Musslick 2021). On the other hand, future studies could assess performance not just once in a posttest but could instead measure it in small steps, that is, several times within the intervention period, in order to get more insight into what really happens during the use of such a learning program. Still, the finding that practice behavior had no effects on addition performance but it did on subtraction performance might also be due to subtraction being more difficult than addition (Anderson et al. 2022; Kamii et al. 2001). Possibly, this is why practicing with Math Garden was particularly helpful for the subtraction domain. However, surprisingly, we found an effect only for the number of subtraction tasks practiced and not for the number of practiced weeks of subtraction. Given the assumptions about distributed learning behavior, which means that practice is most effective when practice sessions are spaced out over time with breaks in between rather than when tasks are repeated in immediate succession (i.e., massed practicing; Carpenter et al. 2012; Gerbier and Toppino 2015; Toppino and Gerbier 2014), it is reasonable to assume that the practiced weeks are a relevant indicator. Missing effects might be because most students practiced only for a small number of weeks and, thus, only little—and probably not meaningful—variance was created within the variables for the practiced weeks. Therefore, future research might systematically vary the practiced weeks, for instance, by reminding a subpopulation of students every day to practice for five minutes. Further, future research might combine the practiced weeks with the practiced tasks measure because, with the current approach, for instance, a student who practiced 100 tasks in four weeks has the same score as a student who practiced 10 tasks in four weeks. Thus, in terms of challenging the hypotheses on massed and distributed practice behavior, future studies might investigate a moderation between the practiced weeks and the practiced tasks. As showing such an interaction effect would require even higher statistical power (e.g., through a sufficiently large sample size; Aiken and West 1991), we concentrated on considering the practiced tasks and practiced weeks separately as direct effects. Additionally, our results indicate that both measures were highly correlated. Hence, suppressor effects might become a problem for future studies investigating such an approach. Thus, we recommend ensuring not only that there are students who practice few tasks in few weeks and many tasks in many weeks but also that there are students who practice few tasks in many weeks and many tasks in few weeks. Still, as no previous study ever took the measure of practiced weeks into account, our approach provides initial, valuable information on how to employ a new practice behavior measure regarding math learning programs.

### 4.2. Effects of Math Garden on Math Self-Concept

Our results suggest that, as expected, providing students with Math Garden fostered their math self-concept in contrast to a wait-list control condition. Considering the fact that math self-concept is one of the most important predictors of later performance (Eccles and Wigfield 2020; Guay et al. 2003; Marsh and Martin 2011), this is a very promising result. Interestingly, the provision of Math Garden in our study seemed to have a buffering effect. While we descriptively observed that math self-concept in the wait-list control condition declined over the intervention period, which is in line with previous research that suggested an overall decrease in self-concept over a school year (Eshel and Klein 1981; de Fraine et al. 2007), the provision of Math Garden appeared to counteract this decline and also led to a significant increase in math self-concept. Thus, students seemed to benefit from Math Garden regarding their math self-concept, which is why we can recommend implementing the program in classrooms. Further, this result is also in line with earlier research: In a Dutch sample, Jansen et al. (2013) showed a small but positive effect of providing Math Garden on perceived math competence, which is a construct that is closely related to math self-concept (Lee 2009). However, they only had an intervention duration of 11.1 weeks on average, and their effect can thus be assumed to be modest because of the short intervention period (Jansen et al. 2013). By replicating this result in a German sample with an intervention duration almost twice as long, our finding supports its robustness. Further, keeping in mind that the students in our sample all came from non-academic-track schools, it is reasonable to assume that they were mostly lower achievers. Although research results have already shown that math learning program interventions can be especially effective for low-achieving students regarding performance (Hassler Hallstedt et al. 2018; Ran et al. 2021), effects on affective-motivational outcomes have not yet been considered. However, as low-achieving students, in particular, might have unfavorable motivational prerequisites after continuous experiences of failure, future research should also focus on investigating such effects in this subpopulation. The results of our study might be the first indication that such interventions have the potential to foster low-achieving students’ math self-concept, which is promising and thus needs further investigation.

Contrary to our expectations, we did not find an effect of practice behavior on math self-concept. This result is in line with research by Jansen et al. (2013), who were not able to show any effects on the construct of perceived math competence, which is related to math self-concept when considering the practiced tasks in Math Garden. Given the positive effects of providing Math Garden on students’ math self-concept, one might conclude that the amount of feedback students receive when practicing with the program does not influence the extent to which the program fosters their math self-concept. An explanation for this missing effect of practice behavior might be found in the study results of Bernacki et al. (2015), who investigated the effect of feedback on the motivational construct of self-efficacy. They showed that feedback on the correctness of tasks affected self-efficacy, especially at the beginning of the training session. However, over time, the previous self-efficacy became more and more predictive of further self-efficacy, while the number of correct answers, and thus positive feedback, became less important. The authors postulated that positive feedback has a positive effect, especially at the start of a learning activity, but that self-efficacy then stabilizes (Bernacki et al. 2015). This might be the same with math self-concept. To test such an assumption for practice behavior with Math Garden, future research that assesses math self-concept multiple times during the intervention period is needed.

### 4.3. Effects of Math Garden on Math Anxiety

We did not find any effects of providing Math Garden or of practice behavior with Math Garden on math anxiety. These missing findings are in line with research from Jansen et al. (2013), who were also not able to show any effects on math anxiety. Regarding these missing effects, research by Hilz et al. (2023) showed that math-anxious students generally practice less with math learning programs. Thus, math-anxious students might not have benefited from the adaptive algorithm as they practiced too little to notice that the program adjusted tasks to their ability level. In turn, they might not have perceived tasks in Math Garden to be controllable, which is why math anxiety did not decrease. Furthermore, many authors have pointed out that math anxiety is a phenomenon that begins at a young age and is, therefore, deeply rooted in students (Ashcraft and Krause 2007; Bekdemir 2010; Ramirez et al. 2013). Therefore, although students’ experiences with Math Garden in the present investigation might have been positive due to the adaptive algorithm and, therefore, a sufficient amount of positive feedback might have been received, these experiences may not outweigh negative math experiences in the past (Jansen et al. 2013). Additionally, our math anxiety items mainly focused on math test anxiety, which is only one dimension of math anxiety. Research has already shown that math anxiety has other facets and components, such as an affective and a cognitive component (Wigfield and Meece 1988), or a trait and a state component (Orbach et al. 2019). Again, to get a more detailed view of the underlying effects of math learning programs, future investigations might include these different components in their investigations. Future research might also experimentally investigate in more detail whether the manipulation of specific program elements has the potential to reduce math anxiety. For example, the developers of Math Garden recently implemented the option of hiding coins when students solve the tasks. Normally, while a specific task is being presented, coins spilling out under the task symbolize the remaining time to give an answer. The more quickly students give the correct answer, the more remaining coins they receive as a reward. However, these coins might put pressure on math-anxious students in particular because working memory capacities might be even more reduced (Ashcraft and Kirk 2001; Hunt and Sandhu 2017). Hence, turning off this visibly expiring time might have a positive effect on students’ math anxiety.

### 4.4. Limitations

Even though our study provides new insights into how math learning programs like Math Garden potentially affect students’ performance, their math self-concept, and their math anxiety, our analyses have limitations that need to be considered when interpreting the results. As already mentioned, the posttest sample size was reduced due to school closures because of the COVID-19 lockdown. Even though this sample dropout appeared to be unsystematic, as we did not suggest testing dates to schools with any specific strategy, and the two subsamples did not differ in their pretest scores, the consequence was a loss of power according to our G*Power analyses (Faul et al. 2007, 2009). Hence, for replicating the robustness of our desirable finding on math self-concept, we recommend replicating our findings in a larger sample.

Due to the fact that most high-achieving students in Germany attend academic-track schools, our sample, which only included non-academic-track schools, consisted of, on average, lower-achieving students. Therefore, the results cannot be generalized to all types of German schools. In turn, as we also did not explicitly consider low achievement, our results can also not be fully generalized to all low-achieving students.

Further, we did not collect information about the environment (i.e., at home or in school) in which students practiced with Math Garden. We assume that using Math Garden voluntarily at home might be more effective regarding performance than using Math Garden during math lessons at school because using Math Garden outside of school means extra learning time. For instance, Bakker et al. (2015) showed that their math learning program intervention was most effective when students practiced at home after being introduced to the program at school. Future research might investigate whether students benefit more from extra practice time at home in contrast to practice time during math lessons.

Moreover, school, teacher, and parental characteristics might have affected our outcome variables. For instance, schools vary in their digital infrastructure and, thus, in the availability of digital devices (Eickelmann et al. 2019; Stanat et al. 2022). Because we randomized students on the class level, the variance between schools should have been controlled for. However, because of the design of our study, we were only able to control for variance differences between classes. Hence, we were unable to consider teacher variables at Level 2 to investigate variance between classes. However, as teachers vary in their competencies and attitudes towards the use of digital media in the classroom (Eickelmann et al. 2019), it is reasonable to assume that such characteristics might have affected our outcomes as well. Further, it would be interesting to know the extent to which teachers used Math Garden during their math instruction. Moreover, we were also not able to take parental characteristics into account. The results of previous research on the quality dimensions of parental homework involvement have already shown that, for instance, high parental control inhibits students’ academic functioning and achievement (Guill et al. 2020; Moroni et al. 2015). Further, we did not know what type of digital devices students used to practice with Math Garden at home. As research on the motives for using digital devices has already shown that, for instance, smartphones are often used for entertainment-related and social-interactive purposes, whereas desktop computers offer better possibilities for information and learning-orientated use (Napoli and Obar 2014), we assume that students might be more distracted when using Math Garden on smartphones instead of on desktop computers. This might have affected their performance. Future research might take such variables into account.

Finally, this study had two design limitations. First, we only investigated the effects of Math Garden compared to a “business as usual” group (i.e., students receiving regular math instruction) that did not receive any support in the form of additional learning opportunities. Thus, the effects of Math Garden can only be interpreted compared to the effects of not having any additional learning opportunities. Thus, the current study rather provides evidence for the effects of Math Garden than for the effectiveness of Math Garden. For the latter, a comparison with another support strategy or other additional learning opportunities with the same invested learning time would be helpful to examine if Math Garden is more effective in supporting learners than other support strategies. Second, randomization took place on the class level rather than on the individual level. This is standard practice when implementing a treatment in educational research to ensure the acceptance and usefulness of the study (Plewis and Hurry 1998), but it should, nonetheless, be kept in mind when interpreting the results.

### 4.5. Practical Implications

Although only some of our theoretical assumptions were confirmed, this study has implications for practitioners who plan to implement a math learning program in the classroom. The distinct performance measure approach could help teachers to decide which program to use in the classroom or educational administrations to decide which specific programs to recommend for classroom use because this approach can clarify which program is effective in fostering a particular math ability (Ran et al. 2021). Thus, with the results of our study, we can recommend that teachers use Math Garden as it might foster their students’ subtraction skills more than if no additional learning opportunities are provided. Further investigations might also test how Math Garden affects students’ multiplication and division skills. However, our study also showed that simply providing the program did not affect students’ performance. Thus, teachers need to actively encourage their students to practice with Math Garden in order for them to benefit regarding their performance. This seems to apply in particular to math-anxious students (Hilz et al. 2023). For instance, teachers might use Math Garden at the end of every math lesson to get their students used to practicing, or they might use it in supervision classes or assign practice as homework. Such strategies should be followed, especially after a vacation period, as students tend to stop practicing during vacations (Hilz et al. 2023; Hofman et al. 2018). Moreover, as Math Garden has the potential to foster students’ math self-concept, the use of Math Garden in the classroom might motivate students to further deal with math and math-related topics, which, in turn, might have long-term effects on future performance (Eccles and Wigfield 2020; Guay et al. 2003; Marsh and Martin 2011).

## 5. Conclusions

Even though research on math learning programs has taken off in recent years (Higgins et al. 2019; Hillmayr et al. 2020; Pellegrini et al. 2021; Sailer and Homner 2020), the results of their effectiveness are heterogeneous, and the programs seem to largely fail to meet the expectation that they can fundamentally revolutionize the school system (Pellegrini et al. 2021). However, instead of concluding that they are ineffective and potentially rejecting their use, this study offers new ways in which future research might address math learning program evaluations and thus adds a new perspective to the current state of research in this field. Our results suggest that the learning program Math Garden might foster students’ subtraction skills more than when an additional learning program is not used, but only when practice behavior is considered. Further, we were able to show that providing Math Garden fosters students’ math self-concept. For future investigations, we would like to encourage researchers to evaluate (math) learning programs on a more detailed level to uncover potentially hidden effects. Thus, we recommend that researchers take the following points into account:

- Focus on measuring distinct subdomains of performance: This can help practitioners, in particular, to make decisions about the target group for whom the program might be most beneficial.
- Take affective-motivational variables into account: Even if a program has no effect on performance shortly after the intervention, performance might increase in the long term if affective-motivational variables, which are predictors of performance, are affected by the intervention. Again, it is also important to investigate single dimensions of these variables in more detail, for instance, to differentiate between the cognitive and the affective component of math anxiety.
- Consider practice behavior with log and trace data: This can provide a deeper insight into the optimal amount of practice with (math) learning programs and which types of students’ behavior might benefit the most from the implementation (e.g., distributed practicing).

## Figures and Tables

**Table 1:** Descriptives of self-report and performance measures.

	All Students *N* = 370			Condition				
				Wait-List Control *n* = 170			Experimental *n* = 200	
	*M*	*SD*		*M*	*SD*		*M*	*SD*
Math addition performance T_1_	5.61	3.12		5.74	3.23		5.49	3.05
Math addition performance T_2_	7.90	2.77		7.83	2.91		7.97	2.66
Math subtraction performance T_1_	5.20	3.63		5.41	3.58		5.01	3.67
Math subtraction performance T_2_	6.62	3.69		6.71	3.53		6.56	3.85
Math self-concept T_1_	2.70	.82		2.67	.84		2.72	.81
Math self-concept T_2_	2.73	.83		2.59	.86		2.85	.78
Math anxiety T_1_	3.36	1.21		3.28	1.22		3.44	1.21
Math anxiety T_2_	3.20	1.26		3.17	1.22		3.23	1.30
Gender ^a^	.49	.50		.47	.50		.50	.50
Migration background T_1_^b^	.49	.50		.43	.50		.53	.50
Tablet typing speed T_1_	7.88	2.36		8.12	2.27		7.66	2.43

*Note*. Reference categories: ^a^ male. ^b^ no other languages spoken at home besides German.

**Table 2:** Descriptives of practice behavior in Math Garden for the experimental condition.

	*M*	*SD*	Range
Addition tasks practiced	177.81	268.08	0–2412
Subtraction tasks practiced	54.63	120.78	0–1369
Overall tasks practiced	1090.91	1366.94	11–8406
Practiced weeks of addition	2.74	2.12	0–10
Practiced weeks of subtraction	1.72	1.67	0–12
Overall weeks practiced	4.62	2.96	1–13

*Note*. Experimental condition only. *n* = 200.

**Table 3:** Correlation matrix of the study variables.

	1	2	3	4	5	6	7	8	9	10	11
1. Math Learning Program ^a^											
2. Math addition performance T_1_	−.05										
3. Math subtraction performance T_1_	−.07	**.69**									
4. Math self-concept T_1_	.03	**.20**	**.32**								
5. Math anxiety T_1_	.07	**−.18**	**−.26**	**−.45**							
6. Gender ^b^	.03	−.00	−.08	**−.27**	**.22**						
7. Migration background T_1_^c^	.10	−.04	−.04	−.04	.01	.06					
8. Tablet typing speed	−.10	**.51**	**.36**	**.21**	−.06	.02	−.05				
9. Math addition performance T_2_	−.03	**.52**	**.57**	**.29**	**−.27**	−.07	−.01	**.31**			
10. Math subtraction performance T_2_	−.05	**.46**	**.62**	**.25**	**−.23**	**−.14**	−.01	**.21**	**.67**		
11. Math self-concept T_2_	**.12**	**.22**	**.34**	**.64**	**−.36**	**−.25**	−.01	**.14**	**.36**	**.35**	
12. Math anxiety T_2_	.05	−.11	**−.21**	**−.32**	**.37**	**.27**	**.16**	.03	**−.19**	**−.19**	**−.45**

*Note*. Correlations refer to all students, *N* = 370. Significant correlations are printed in bold (*p* ≤ .05). Reference categories: ^a^ wait-list control condition. ^b^ male. ^c^ no other languages spoken at home besides German.

**Table 4:** Correlation matrix for practice behavior and outcomes.

	1	2	3	4	5	6	7	8	9
1. Addition tasks practiced									
2. Subtraction tasks practiced	**.36**								
3. Overall tasks practiced	**.69**	**.40**							
4. Practiced weeks of addition	**.71**	**.49**	**.63**						
5. Practiced weeks of subtraction	**.52**	**.77**	**.52**	**.77**					
6. Overall weeks practiced	**.48**	**.46**	**.72**	**.76**	**.65**				
7. Math addition performance T_2_	−.12	−.00	−.01	.01	−.06	.09			
8. Math subtraction performance T_2_	−.07	.02	−.03	.03	−.00	.07	**.71**		
9. Math self-concept T_2_	.02	.05	−.01	−.05	−.01	−.02	**.20**	**.29**	
10. Math anxiety T_2_	−.07	−.07	−.01	−.01	.03	.04	−.08	−.14	**−.52**

*Note.* Correlations refer to the experimental condition only, *n* = 200. Significant correlations are printed in bold (*p* ≤ .05).

**Table 5:** Results on effects of providing Math Garden.

	Addition Performance				Subtraction Performance				Math Self-Concept				Math Anxiety		
	β	*SE*	*p*		β	*SE*	*p*		β	*SE*	*p*		β	*SE*	*p*
*Predictors*															
Outcome at T_1_	**.49**	.10	.000		**.61**	.06	.000		**.61**	.05	.000		**.35**	.05	.000
Math Learning Program ^a^	−.00	.07	.960		−.02	.07	.727		**.12**	.04	.002		.01	.07	.883
*Covariates*															
Gender ^b^	−.07	.05	.255		−.09	.07	.184		−.08	.07	.212		**.18**	.08	.031
Migration background T_1_^c^	−.01	.06	.907		.02	.06	.744		−.00	.05	.957		**.16**	.07	.034
Tablet typing speed T_1_	.08	.15	.469		−.02	.07	.813		.04	.08	.633		.05	.05	.256
*R* ^2^	.29				.38				.43				.21		

*Note*. Significant coefficients are printed in bold (*p* ≤ .05). Continuous predictors were standardized (*M* = 0; *SD* = 1). *n* = 370. Reference categories: ^a^ wait-list control condition. ^b^ male. ^c^ no other languages spoken at home besides German.

**Table 6:** Results on effects of practice behavior.

	Addition Performance				Subtraction Performance				Math Self-Concept				Math Anxiety		
	β	*SE*	*p*		β	*SE*	*p*		β	*SE*	*p*		β	*SE*	*p*
PRACTICED TASKS															
*Predictors*															
Outcome at T_1_	**.42**	.13	.001		**.65**	.07	.000		**.54**	.07	.000		**.37**	.08	.000
Practiced tasks	.03	.09	.767		**.10**	.04	.008		-.01	.10	.931		.08	.11	.455
*Covariates*															
Gender ^a^	−.10	.08	.205		−.14	.10	.152		−.20	.11	.070		.21	.13	.114
Migration background T_1_^b^	−.03	.06	.593		.08	.07	.278		−.09	.08	.242		**.25**	.11	.027
Tablet typing speed T_1_	.23	.15	.128		.03	.09	.746		.00	.12	.990		.01	.07	.865
*R* ^2^	.31				.48				.41				.25		
PRACTICED WEEKS															
*Predictors*															
Outcome at T_1_	**.42**	.12	.001		**.63**	.08	.000		**.54**	.07	.000		**.37**	.07	.000
Practiced weeks	.11	.06	.081		.05	.05	.363		−.01	.06	.815		.11	.07	.113
*Covariates*															
Gender ^a^	−.11	.07	.123		−.15	.09	.107		−.20	.11	.068		.21	.13	.105
Migration background T_1_^b^	−.03	.06	.589		−.08	.08	.316		−.09	.08	.282		**.25**	.12	.038
Tablet typing speed T_1_	.24	.16	.118		.04	.09	.635		.00	.13	.988		.01	.07	.935
*R* ^2^	.33				.47				.41				.26		

*Note*. Significant coefficients are printed in bold (*p* ≤ .05). Continuous predictors were standardized (*M* = 0; *SD* = 1). *n* = 200. Reference categories: ^a^ male. ^b^ no other languages spoken at home besides German.

## Data Availability

Data are available upon request.

## Supplementary Materials

The following supporting information can be downloaded at: https://www.mdpi.com/article/10.3390/jintelligence11060108/s1, Table S1: Student descriptives of performance items >.30 item-scale correlation; Table S2: Student descriptives of all performance items; Table S3: Effects of practice behavior on performance with items >.30 item-scale correlation; Table S4: Effects of practice behavior on performance with all performance items; Table S5: Comparing study variables with and without missing values.
