# Supplementary material for: How to Continue? New Approaches to Investigating the Effects of Adaptive Math Learning Programs on Students’ Performance, Self-Concept, and Anxiety
Source: J Intell. 2023 Jun 1;11(6):108. doi: 10.3390/jintelligence11060108 (PMC10299571; doi:10.3390/jintelligence11060108)
Supplement: Supplementary file 1 [file jintelligence-11-00108-s001.zip › jintelligence-2319446-supplementary.pdf]

Journal of *Intelligence*

MDPI

Supplementary Materials

Table S1. Effects of providing Math Garden on performance with items > .30 item-scale correlation

	Addition performance			Subtraction performance		
	β	*SE*	*p*	β	*SE*	*p*
Predictors						
Outcome at T_1	**.55**	.10	.000	**.67**	.06	.000
Math Learning Program[a]	–.00	.07	.973	–.01	.07	.941
Covariates						
Gender[b]	–.08	.05	.162	–.09	.05	.117
Migration background T_1[c]	.02	.05	.709	.04	.05	.425
Tablet typing speed T_1	.05	.09	.631	–.02	.06	.769
R^2		.33			.46	

Note. Significant coefficients are printed in bold ($p \leq .05$). Continuous predictors were standardized ($M = 0$; $SD = 1$). $n = 370$. Reference categories: [a] wait-list control condition. [b] male. [c] no other languages spoken at home besides German.

Table S2. Effects of providing Math Garden on performance with all performance items

	Addition performance			Subtraction performance		
	β	*SE*	*p*	β	*SE*	*p*
Predictors						
Outcome at T_1	**.55**	.10	.000	**.65**	.06	.000
Math Learning Program[a]	.03	.07	.686	–.01	.07	.918
Covariates						
Gender[b]	–.07	.05	.148	–.08	.06	.134
Migration background T_1[c]	–.05	.05	.296	.05	.05	.249
Tablet typing speed T_1	.02	.08	.788	.01	.06	.912
R^2		.32			.45	

Note. Significant coefficients are printed in bold ($p \leq .05$). Continuous predictors were standardized ($M = 0$; $SD = 1$). $n = 370$. Reference categories: [a] wait-list control condition. [b] male. [c] no other languages spoken at home besides German.

Table S3. Effects of practice behavior on performance with items > .30 item-scale correlation

	Addition performance			Subtraction Performance		
	β	*SE*	*p*	β	*SE*	*p*
PRACTICED TASKS						
Predictors						
Outcome at T_1	**.49**	.14	.000	**.70**	.06	.000
Practiced tasks	.07	.07	.316	**.12**	.05	.015
Covariates						
Gender[a]	–.11	.06	.056	–.14	.10	.095
Migration background T_1[b]	–.00	.05	.976	–.04	.06	.515
Tablet typing speed T_1	.16	.12	.185	–.01	.08	.885
R^2		.34			.53	
PRACTICED WEEKS						
Predictors						
Outcome at T_1	**.49**	.14	.000	**.68**	.07	.000
Practiced weeks	**.13**†	.05	.015	.06	.06	.282
Covariates						
Gender[a]	**–.13**†	.05	.014	**–.15**†	.07	.045
Migration background T_1[b]	–.00	.05	.953	–.04	.06	.589
Tablet typing speed T_1	.18	.12	.154	–.01	.09	.928
R^2		.36			.52	

Note. Significant coefficients are printed in bold ($p \leq .05$). Continuous predictors were standardized (M = 0; SD = 1). n = 200. Reference categories: [a] male. [b] no other languages spoken at home besides German. †Coefficient differed in significance level ($p \leq .05$) in contrast to original analysis with item-scale correlation > .5.

Table S4. Effects of practice behavior on performance with all performance items

	Addition performance			Subtraction Performance		
	β	*SE*	*p*	β	*SE*	*p*
PRACTICED TASKS						
Predictors						
Outcome at T_1	**.47**	.14	.001	**.70**	.07	.000
Practiced tasks	.09	.08	.237	**.14**	.04	.001
Covariates						
Gender[a]	**–.11**†	.05	.042	–.13	.08	.093
Migration background T1[b]	.03	.05	.507	–.01	.06	.894
Tablet typing speed T_1	.14	.11	.199	.01	.07	.939
R^2		.30			.54	
PRACTICED WEEKS						
Predictors						
Outcome at T_1	**.47**	.14	.001	**.68**	.07	.000
Practiced weeks	.11	.06	.070	.09	.05	.087
Covariates						
Gender[a]	**–.12**†	.05	.012	**–.15**†	.07	.041
Migration background T1[b]	.03	.06	.568	–.01	.06	.933
Tablet typing speed T_1	.15	.11	.167	.03	.08	.692
R^2		.31			.52	

Note. Significant coefficients are printed in bold ($p \leq .05$). Continuous predictors were standardized ($M = 0$; $SD = 1$). $n = 200$. Reference categories: [a] male. [b] no other languages spoken at home besides German. †Coefficient differed in significance level ($p \leq .05$) in contrast to original analysis with item-scale correlation > .5.

Table S5. Comparing Study Variables With and Without Missing Values

	**Without Missing Values**		**With Missing Values**	
	M	*SD*	*M*	*SD*
ALL STUDENTS				
Math addition performance T_1	5.76	3.15	5.38	3.11
Math addition performance T_2	7.88	2.76	8.40	2.84
Math subtraction performance T_1	5.24	3.74	5.13	3.47
Math subtraction performance T_2	6.62	3.73	6.90	3.00
Math self-concept T_1	2.73	0.86	2.65	0.76
Math self-concept T_2	2.74	0.83	2.36	0.82
Math anxiety T_1	3.39	1.24	3.32	1.12
Math anxiety T_2	3.21	1.28	3.02	1.00
Gender[a]	0.48	0.50	0.50	0.50
Migration background T_1[b]	0.52	0.50	0.44	0.50
Tablet typing speed T_1	7.97	2.20	7.75	2.59
EXPERIMENTAL CONDITION				
Practiced addition tasks	193.36	246.17	159.70	291.88
Practiced subtraction tasks	**69.61**	150.24	**37.19**	69.72
Overall practiced tasks	**1280.06**	1467.16	**870.58**	1211.08
Practiced weeks addition	**3.02**	2.42	**2.42**	1.66
Practiced weeks subtraction	**1.97**	2.01	**1.43**	1.08
Overall practiced weeks	**5.58**	3.31	**3.51**	2.00

Note. All students: $n_{without}$ = 214 , n_{with} = 156. Experimental condition: $n_{without}$ = 116, n_{with} = 84. Bold print indicates values that were statistically significantly different at $p \leq .05$. Reference categories: [a] male. [b] no other languages spoken at home besides German.
